# Assessing the impact of 24-hour activity behaviors on cardiorespiratory fitness in the older adult: a component analysis approach

**DOI:** 10.3389/fpubh.2024.1478533

**Published:** 2024-11-18

**Authors:** Donglei Lu, Wenyu Zhang, Sijie Tan

**Affiliations:** ^1^Tianjin Key Laboratory of Physical and Health Integration and Health Promotion, Tianjin, China; ^2^National Clinical Research Center for Acupuncture and Moxibustion of Traditional Chinese Medicine, Tianjin, China

**Keywords:** physical activity, sleep, sedentary, cardiorespiratory fitness, aging

## Abstract

**Background:**

Cardiorespiratory fitness (CRF) serves as a critical measure of the cardiovascular system’s efficiency in delivering oxygenated blood to tissues and organs. Understanding the relationship between various activity behaviors and CRF is essential for devising effective health interventions for the older adult population.

**Objective:**

This study aimed to investigate the association between 24-h activity behaviors and CRF in older adult individuals, utilizing compositional data analysis.

**Methods:**

We utilized baseline data from the “Fifth National Physical Fitness Monitoring Cohort Study in Tianjin, China,” which included 540 older adult participants. Physical activity and sedentary behavior were objectively measured using a three-dimensional accelerometer, and CRF was assessed via a gas metabolism analyzer. Compositional data analysis was employed to examine the relationships between 24-h activity behaviors—specifically, moderate-to-vigorous physical activity (MVPA), light physical activity (LPA), sedentary behavior (SB), and sleep (SLP)—and CRF.

**Results:**

The analysis demonstrated that MVPA was significantly positively associated with CRF (βMVPA = 5.36, *p* < 0.01), whereas SB was significantly negatively associated (βSB = −3.97, *p* < 0.01). No significant associations were observed for LPA and SLP with CRF. The isochronous substitution model revealed that reallocating 15 min of MVPA to SB, LPA, or SLP significantly decreased CRF by 0.31, 0.27, and 0.23 mL/kg/min, respectively. Conversely, substituting 15 min of SB, LPA, or SLP with MVPA resulted in increases in CRF by 0.29, 0.22, and 0.17 mL/kg/min, respectively. Additionally, replacing SB with LPA or SLP led to improvements in CRF, though these changes were not significant, underscoring the potential benefits of reducing sedentary time and enhancing physical activity levels.

**Conclusion:**

These findings underscore the critical role of increasing MVPA and reducing SB in improving CRF among the older adult. This study provides a robust scientific foundation for health promotion and intervention strategies targeting older adults. Comprehensive modifications to daily activity patterns are imperative for optimizing cardiovascular health in this population.

## 1 Introduction

Cardiorespiratory fitness (CRF) is a critical measure of the cardiovascular system’s efficiency in delivering oxygenated blood to tissues, including skeletal muscle (1). In 2016, the American Heart Association (AHA) designated CRF as the fifth vital sign, underscoring its essential role in clinical evaluations (2). A 2022 statement further emphasized the importance of assessing CRF in older adults to mitigate the increased risk of cardiovascular diseases in this population (3). CRF is particularly vital in the older adult, as it not only reduces the risk of cardiovascular disease (4) but also enhances physical function (5), maintains independence (6), and improves overall quality of life (7). Thus, efforts to preserve and enhance CRF in older adults are paramount in reducing mortality and promoting healthier aging (8).

Despite considerable public health efforts, the burden of cardiovascular diseases among the older adult remains alarmingly high. Between 1990 and 2019, the global prevalence nearly doubled, escalating from 271 million to 523 million cases, with deaths rising sharply from 12.1 million to 18.6 million. This surge is largely attributed to modifiable risk factors, including unhealthy lifestyles, poor dietary habits, and inadequate physical activity (9, 10). The global decline in CRF is traditionally attributed to reduced physical activity (PA) and increased sedentary behavior (SB) (5). However, emerging evidence highlights the critical role of sleep in maintaining CRF. Luo et al. (11) showed that adherence to 24-h movement guidelines, particularly optimal sleep duration, is strongly associated with better CRF and overall health in older adults. Similarly, Liang et al. (12) reported that sufficient sleep is linked to enhanced cardiovascular endurance and blood pressure regulation. These findings suggest that integrating sleep management with PA may be crucial for effective health interventions aimed at preserving cardiovascular health in aging populations (13). Previous research often examined relationships between PA, SB, and CRF in isolation, treating PA and SB as independent factors influencing CRF (14, 15). However, many of these studies failed to acknowledge interconnections among various activity behaviors within a 24-h period (16). A 24-h activity cycle is better understood as a continuum, ranging from inactivity to high intensity, primarily comprising PA, SB, and sleep (17). PA can be further divided into moderate-to-vigorous physical activity (MVPA) and light physical activity (LPA). Scholars have noted that traditional linear regression methods used to analyze 24-h activity behaviors often fail to adequately address collinearity among these behaviors, resulting in a lack of scientific rigor and a holistic perspective (18). The emergence of compositional data analysis offers new insights and methodologies to better address these research challenges.

Compositional data refers to datasets composed of multiple components whose sum is constrained to a constant total, commonly referred to as the “constant sum constraint” (19). In the context of 24-h activity behaviors, this implies that an increase in one type of activity necessarily results in a corresponding decrease in another, given the fixed duration of 24 h. Compositional data analysis effectively addresses this constraint by applying isometric log-ratio (ILR) transformation, which allows for the subsequent use of traditional regression methods to examine the relationships between individual components and health outcomes. Previous studies utilizing compositional data analysis have explored associations between 24-h activity behaviors and various health indicators, including motor skills (20), obesity, CRF, and cardiometabolic health (21).

Despite these advancements, the application of compositional data analysis has predominantly remained theoretical (22), with empirical research largely concentrated on children and adolescents, particularly in areas such as obesity (23), basic motor skills (24), physical fitness (25), and health-related fitness (26). Recent evidence from Liang et al. employed a compositional data analysis to comprehensively evaluate the associations between 24-h activity behaviors and various physical health metrics in older adults. While this study offers valuable insights into these interrelationships, a critical gap remains in understanding the link between 24-h activity behaviors and CRF, particularly with maximal oxygen uptake as the primary outcome measure (27). This study aims to address this gap by focusing on CRF in older adults. We apply compositional data analysis to examine the relationship between 24-h activity behaviors and CRF, and further analyze the anticipated changes in CRF resulting from isotemporal substitution among these behaviors.

## 2 Methods

### 2.1 Participants

This cross-sectional study utilized data from the baseline dataset of the “Fifth National Physical Fitness Monitoring Cohort Study in Tianjin, China.” Designed to explore the relationship between daily physical activity and physical health levels in the older adult, as well as the contributing factors, this cohort study collected data during two distinct periods: February 2022 to July 2023, and February 2023 to July 2024. Informed consent was obtained from all participants following a thorough briefing on the research purpose and significance. Participants were assured that they could withdraw from the study at any point, without any conditions, should they experience discomfort. Approval for this study was granted by the Ethics Committee of Tianjin Sports University (Ethics Committee Code: TJUS2024-029).

A multi-stage hierarchical random sampling method was employed in this study, which considered administrative divisions and community characteristics to select 20 representative communities from six urban areas in Tianjin. To ensure representativeness and consistency, each community provided 30 subjects. The screening criteria required participants to be 60 years or older, to have been permanent residents for over six months, and to be capable of performing daily activities independently. Additionally, signed informed consent was mandatory. From each of the age groups 60–64, 65–69, 70–74, and 75–79, 150 older adult individuals were randomly chosen to maintain a 50% gender balance. After removing invalid data related to maximum oxygen uptake and physical activity, the final dataset included 540 valid samples.

**Figure 1:**
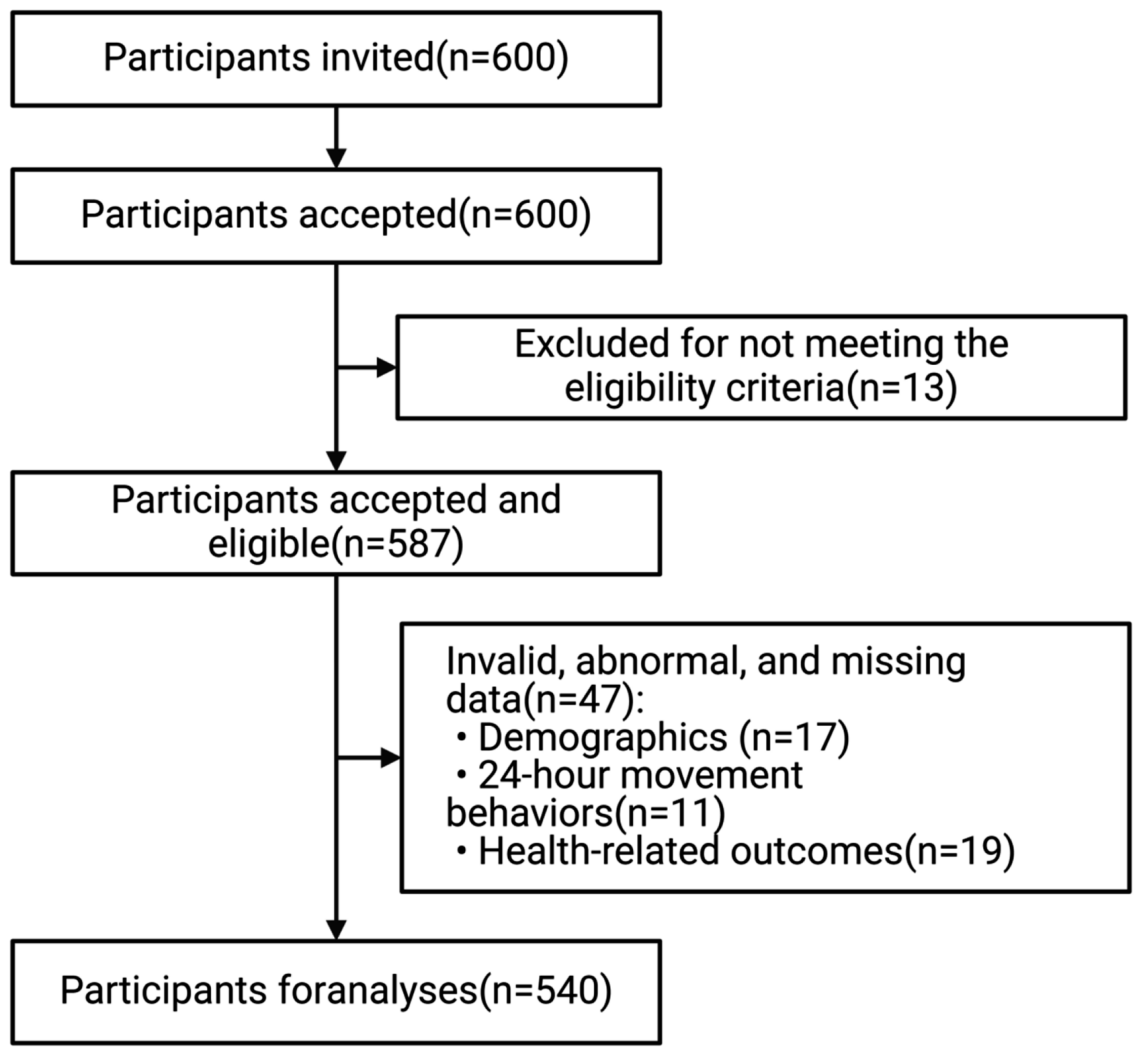
Flowchart of participant recruitment.

Inclusion criteria for subjects were as follows: (1) older adult individuals residing in urban Tianjin, China, and (2) Voluntary participation with informed consent. Individuals were excluded if they met any of the following criteria: (1) Presence of chronic diseases affecting the heart, respiratory system, kidneys, or liver; (2) Severe musculoskeletal or joint diseases, or an inability to participate in exercise experiments due to mental or physical disorders; (3) Inability to comply with the physical activity (PA) data collection requirements, see Figure 1.

### 2.2 Body composition and basic information

#### 2.2.1 Body composition

Body composition of the subjects was measured using dual-energy X-ray absorptiometry (GE Prodigy Lunar DXA). This technique, widely recognized for its accuracy, is commonly employed to assess human body composition. The measurements included total body fat percentage, lean body mass, and bone mineral content. The scanning process was conducted with the subjects in a supine position while maintaining minimal movement to ensure accuracy. Prior to the assessment, subjects were instructed to avoid any vigorous physical activity and fasting for at least 8 h to minimize potential interference with the results. Regional body composition (e.g., arms, legs, trunk) was also analyzed to provide a more detailed assessment. Calibration of the DXA scanner was performed daily using a standard phantom provided by the manufacturer to ensure data consistency and reliability.

**Figure 2:**
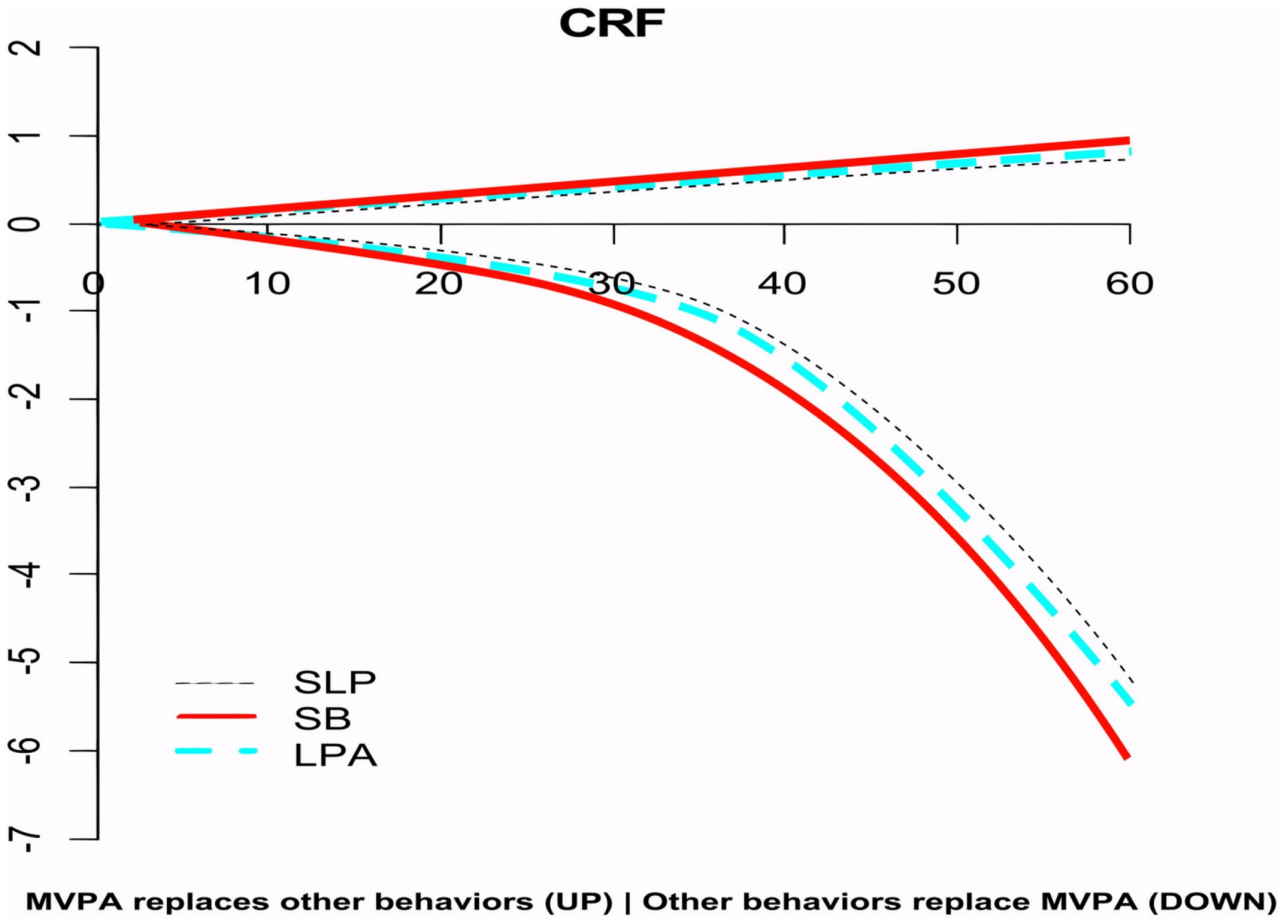
The impact of MVPA equivalent to other behaviors on CRF.

#### 2.2.2 Physical activity and SB measurement

Using a three-dimensional accelerometer (Actigraph GT3X+, United States), activity data were collected over a monitoring period of up to 22 days, capturing physical activity counts, intensity, and duration. The device tracked the older adult participants’ time spent in LPA, moderate-to-vigorous physical activity MVPA, and SB. Worn on the right hip, participants were instructed to keep the accelerometer on for 7 consecutive days, except during bathing, swimming, and sleeping, without altering their daily routines. To determine non-wearing periods, any stretch of at least 60 consecutive minutes with a zero count value was defined, ensuring that the effective wearing time met the 8-h daily standard. The 7-day period covered at least 2 weekdays and 1 weekend day (28).

Activity intensity was classified using the cut-off points defined by Miller et al. (29), categorizing SB as <100 counts/min, LPA as 100–1951 counts/min, and MVPA as ≥1952 counts/min. These classifications were used to determine the duration of activities across different intensity levels. The accelerometer’s parameter settings were carefully aligned with existing studies on older adult physical activity, ensuring consistency in the test instrument, sampling interval, definition of non-wearing time, effective daily wearing time, number of days included in statistical analysis, and cut-off points for physical activity intensity (Table 1).

**Table 1:** Parameter settings for physical activity measurement using a three-dimensional accelerometer.

Parameter content	Parameter settings
test instrument	ActiGraph GT3X+
sampling interval	10s
Not worn time	Choi Algorithm
Effective wearing time per day	≥480 min
Number of days included in effective statistical analysis	At least 3 days (2 working days +1 weekend day)
Physical activity intensity cutoff	
SB/(counts/min)	<100
LPA/(counts/min)	1900–1951
MVPA/(counts/min)	≥1952

SB: sedentary behavior, LPA: low-intensity physical activity, MVPA: moderate-to-high-intensity physical activity.

#### 2.2.3 Cardiopulmonary fitness measurement

To evaluate the maximum oxygen uptake (VO2max) of all participants before and after training, this study utilized a gas metabolism analyzer (Cortex MetaMax 3B), a power bike (Ergoline), a Polar heart rate monitor (chest strap), and a 12-lead ECG monitor (Custo Med). The exercise test required participants to maintain a speed of 60 revolutions per minute while gradually increasing the resistance load on the power bike. For male subjects, the starting load was set at 30 watts (W), increasing by 20 watts per level, with each level lasting 2 min. For female subjects, the starting load was set at 20 watts, increasing by 15 watts per level, with each level also lasting 2 min. Throughout the test, the research team closely monitored the subjects’ 12-lead electrocardiograms, blood pressure changes, gas metabolism indicators, and physical signs, and inquired about their physical sensations.

#### 2.2.4 Covariates

We collected baseline data on gender, age, place of residence, and socioeconomic status (SES) through a self-report questionnaire. SES was assessed using three traditional indicators: education, income, and occupation. Research has shown that combining these indicators into a composite variable can provide a more nuanced and accurate representation of participants’ SES (12, 27). In our study, we used principal component analysis to create a composite SES score for the older adult, focusing on educational attainment, annual *per capita* household income, and accessibility to medical services. The analysis produced a KMO statistic of 0.6, and Bartlett’s test of sphericity was significant (*p* < 0.001), confirming that the selected indicators were suitable for this method. Only one eigenvalue exceeded 1 (1.198), leading to the extraction of a single principal component that explained 53.2% of the variance. We calculated the SES composite score using the formula: SES composite score = 0.471 × annual *per capita* household income +0.566 × educational attainment +0.541 × accessibility to medical services. Previous studies have linked these SES factors to physical activity, SB, and cardiorespiratory fitness. To ensure our findings were robust, we carefully controlled for these potential confounders in our analysis.

#### 2.2.5 Quality control

Prior to conducting any project tests, all testers underwent standardized training and completed assessments to confirm their proficiency in the test methods. Data entry was independently carried out by two testers, with their results cross-checked to detect any discrepancies. Any inconsistencies were immediately verified and corrected, ensuring the accuracy of the data.

### 2.3 Statistical analysis

A total of 60 subjects were excluded for not meeting the inclusion criteria, including: (1) presence of chronic diseases affecting the heart, respiratory, renal, or hepatic systems; (2) severe musculoskeletal or joint disorders, or inability to participate in exercise tests due to mental or physical disabilities; and (3) inability to adhere to PA data collection requirements. Ultimately, 540 valid samples were retained for analysis. Descriptive statistics, including mean, standard deviation, frequency, and percentage, were used to summarize sample characteristics. Physical activity behavior was characterized using central tendency (geometric means of each behavior adjusted to total 1,440 min) and dispersion (variation matrix summarizing data structure via log-ratio variances of all behavior pairs). Values in the combined variation matrix close to zero indicate high correlations between two behaviors, suggesting they are easily reallocated.

For data analysis, we first performed outlier detection using Cook’s Distance and Leverage tests, identifying 12 potential outliers. Robust regression revealed that eight had minimal impact on the model and were retained, while four significant outliers were excluded to ensure data integrity.

The Shapiro–Wilk test indicated that most variables, including SB, MVPA, and LPA (*p* = 0.001, 0.004, and 0.002, respectively), deviated from a normal distribution. After log transformation, *p*-values improved to 0.210, 0.315, and 0.254, meeting the criteria for normality.

Approximately 5% of the data, mainly related to physical activity and sleep duration, were missing. Multiple imputation with Predictive Mean Matching was used to generate five complete datasets. Post-imputation analysis confirmed high consistency with the original data, validating the effectiveness of the imputation method.

For data analysis, we adhered to the “Guidelines for Component Data Analysis” as outlined by Chastin et al. (16). Given that the collected data did not encompass a full 24-h period, we employed the “closed method” to standardize the data (27). The data processing was anchored on the measured sleep duration. The specific formula used is (Equations 1–13):


(1) $$d_{x}=\left(\frac{d}{B_{SB}+A_{\mathrm{MVPA}}+A_{LPA}}\right)\times \left(1440-S\right)$$

Where: d is the measured value of a specific behavior (SB, MVPA, or LPA), *B_SB_* is the SB value, *AM_VPA_* is the MVPA value, *A_LPA_* is the LPA value, and *S* is the sleep time.

1. The component geometric mean shows the central trend of the 24-h activity behavior, and the variance matrix (i.e., the paired equal-interval logarithmic ratio variance) reflects the discrete situation of the 24-h activity behavior, such as the variance of ln (*A_MVPA_/S*). A variance close to 0 indicates a high degree of interdependence between the corresponding two behaviors, while a variance closer to 1 indicates lower interdependence.
2. Apply the equal-interval logarithmic ratio transformation to each activity behavior according to the formula (16).
3. Use the ILR transformation to convert the component data as per the specified formula.

Transformation 1:


(2) $$z_{1}=\sqrt{3/4}\;\ln\frac{A_{\mathrm{MVPA}}}{\sqrt[{3}]{S\times A_{LPA}\times B_{SB}}}$$

(3) $$z_{2}=\sqrt{2/4}\;\ln\frac{S}{\sqrt[{2}]{A_{LPA}\times B_{SB}}}$$

(4) $$z_{3}=\sqrt{1/4}\;\ln\frac{B_{SB}}{\sqrt[{1}]{A_{LPA}}}$$

Transformation 2:


(5) $$z_{1}=\sqrt{3/4}\;\ln\frac{S}{\sqrt[{3}]{A_{LPA}\times B_{SB}\times A_{\mathrm{MVPA}}}}$$

(6) $$z_{2}=\sqrt{2/4}\;\ln\frac{B_{SB}}{\sqrt[{2}]{A_{LPA}\times A_{\mathrm{MVPA}}}}$$

(7) $$z_{3}=\sqrt{1/4}\;\ln\frac{A_{LPA}}{\sqrt[{1}]{A_{\mathrm{MVPA}}}}$$

Transformation 3:


(8) $$z_{1}=\sqrt{3/4}\;\ln\frac{B_{SB}}{\sqrt[{3}]{A_{LPA}\times S\times A_{\mathrm{MVPA}}}}$$

(9) $$z_{2}=\sqrt{2/4}\;\ln\frac{B_{SB}}{\sqrt[{2}]{S\times A_{\mathrm{MVPA}}}}$$

(10) $$z_{3}=\sqrt{1/4}\;\ln\frac{A_{\mathrm{MVPA}}}{\sqrt[{2}]{S}}$$

Transformation 4:


(11) $$z_{1}=\sqrt{3/4}\;\ln\frac{A_{LPA}}{\sqrt[{3}]{S\times B_{SB}\times A_{\mathrm{MVPA}}}}$$

(12) $$z_{2}=\sqrt{2/4}\;\ln\frac{A_{\mathrm{MVPA}}}{\sqrt[{2}]{S\times B_{SB}}}$$

(13) $$z_{3}=\sqrt{1/4}\;\ln\frac{S}{\sqrt[{1}]{B_{SB}}}$$

Following the ILR transformation, the four component data were used as independent variables, with CRF serving as the dependent variable in the component data analysis. The component regression results reflect the relationship between the proportion of time spent on specific activity behaviors (as opposed to absolute time) relative to the time spent on other behaviors and CRF. Based on the fitted component regression model, time was reallocated in 15-min increments to estimate the expected change in CRF (30). This approach involved reallocating 15 min from one activity behavior to another—for example, increasing MVPA time while reducing SB time—while keeping the total time (1,440 min) and the time allocated to other activities, such as LPA and sleep, constant.

Previous studies have demonstrated that even a 15-min modification in activity behavior can markedly influence health outcomes (27). In this study, we initially reallocated 15 min from one activity to another to estimate the resultant changes in outcome indicators via isochronous substitution. To ensure methodological consistency with prior research, we extended this approach by performing “one-to-one” isochronous substitutions in 5-min increments, progressively extending up to 60 min (27).

This study applied the compositional data analysis method introduced by Chastin et al. (16) and conducted the statistical analysis using the *compositions* package in R version 4.2.3. By following these established guidelines, we ensured rigorous analysis of the compositional data.

## 3 Results

### 3.1 Descriptive results analysis

A total of 540 participants were included in the study, comprising 252 males (46.67%) and 288 females (53.33%), with a mean age of approximately 65 years. Statistical analysis revealed significant differences in height, weight, and BMI between male and female older adult participants (*p* < 0.05). In contrast, socioeconomic status did not significantly differ between genders (Table 2).

**Table 2:** Descriptive characteristics of the study sample (*n* = 540).

Variable	Total (*n* = 540)
Age (yr), mean(SD)	65.69 ± 5.53
Gender, *n*(%)	
Male	252(46.7)
Female	288(53.3)
Residence, *n*(%)	
Urban	265(49.1)
Countryside	275(50.9)
Education, *n*(%)	
Primary school and blow	235(43.5)
Secondary school	193(35.8)
College and above	112(20.7)
Occupation	
Manual labor	306(56.7)
Mental labor	234(43.3)
Income	
<3,000	315(58.4)
≥3,000	225(41.6)
Marital status, *n*(%)	
Married	453(83.9)
Single/divorced/widowed	87(16.1)
Chronic disease, *n*(%)	
No	101(18.7)
Yes	439(81.3)
Smoking, *n*(%)	
Not currently	92(17.0)
Yes, but not everyday	46(8.6)
Yes, almost everyday	402(74.4)
Alcohol, *n*(%)	
Yes	363(67.2)
No	177(32.8)
Physical outcome	
Height (cm)	162.4(7.6)
Weight (kg)	67.4(9.3)
BMI (kg/m2)	25.5(3.2)

### 3.2 Characteristics of component variables

Older adult individuals predominantly allocate their time to SB and SLP. The geometric means for MVPA, LPA, SB, and SLP are 3.27, 11.39, 45.27, and 41.07%, respectively (Table 3). Analysis of the variation matrix indicates a high degree of interdependence between SB and SLP (ln SB/SLP = 0.11), suggesting that these activities are more readily interchangeable. In contrast, the interdependence between MVPA and SB is notably lower (ln MVPA/SB = 0.47), indicating a reduced likelihood of these behaviors substituting for one another.

**Table 3:** Central tendency and dispersion of component data.

	MVPA	LPA	SB	SLP
Component mean	3.27	11.39	45.27	4 0 0.0 6
Mean value of ingredients (min/day)	47.09	164.02	651.89	576.86
MVPA	0	0.37	0.47	0.41
LPA	0.37	0	0.25	0.39
SB	0.47	0.25	0	0.11
SLP	0.41	0.39	0.11	0

### 3.3 Component regression of 24-h activity and CRF in the older adult

After adjusting for covariates such as BMI and SES, the ILR-transformed 24-h activity behaviors (MVPA, LPA, SB, and SLP) were analyzed as independent variables, with CRF as the dependent variable, using the component analysis method (Table 4). Across all four models, the *p* value, R^2^, intercept, and covariates remained consistent (CRF: *p* < 0.001, R^2^ = 0.12). The component regression model revealed a significant positive correlation between the proportion of time spent on MVPA (βMVPA = 5.36, *p* < 0.01) and CRF, indicating that as MVPA time increased—at the expense of LPA, SB, and SLP time—CRF improved. Conversely, the proportion of time allocated to sedentary behavior (βSB = −3.97, *p* < 0.01) was significantly negatively correlated with CRF, suggesting that increased SB time—relative to MVPA, LPA, and SLP—led to a decline in CRF. No significant correlations were observed between LPA, SLP, and CRF.

**Table 4:** Component linear regression of 24-h activity behavior time distribution and CRF.

Activity Behavior	β	*P*	Model P value	Std. error	*t*	Model R ^2^
ILR MVPA/(SLP*SB*LPA)	5.36	0.01	<0.001	3.760	14.374	0.12
ILR LPA/(SLP*SB*MVPA)	2.73	0.28				
ILR SB/(SLP*LPA*MVPA)	−3.97	0.01				
ILR SLP/(SB*LPA*MVPA)	−1.28	0.62				

The above model adjusted for age, sex, place of residence, BMI, and SES; the β value represents the effect of a given behavioral change on CRF relative to other behaviors, such as ILR MVPA/(SLP*SB*LPA) refers to the effect of a change in MVPA on CRF relative to SLP, SB, and LPA.

### 3.4 Isochronous substitution model of 24-h activity and CRF

To assess the effects of isochronous substitution on CRF, we reallocated 15 min from one activity to another and analyzed the resulting changes. When 15 min of MVPA was substituted with SB, LPA, or SLP, CRF increased by 0.29, 0.22, and 0.17 mL/kg/min, respectively. Conversely, replacing 15 min of SB, LPA, or SLP with MVPA led to a decrease in CRF by 0.31, 0.27, and 0.23 mL/kg/min, respectively. Additionally, substituting 15 min of SLP with SB resulted in a CRF increase of 0.09 mL/kg/min. However, replacing SB with LPA or SLP caused a decrease in CRF by 0.18 and 0.11 mL/kg/min, respectively. These results, detailed in Table 5, underscore the significant impact of activity reallocation on cardiorespiratory fitness.

**Table 5:** Changes in 15-min isochronous substitution and CRF prediction values for 24-h activity behavior.

	SB ↓	LPA ↓	MVPA ↓	SLP ↓
SB ↑		−0.1 8 *	−0.31*	−0.1 1 *
LPA ↑	0.08		−0.27*	0.12
MVPA ↑	0.29*	0.22*		0.17*
SLP ↑	0.09*	−0.17	−0.23*	

Age, gender, place of residence, BMI and SES were adjusted in the model; ↑ represents an increase of 15 min in the duration of the activity, ↓ represents a decrease of 15 min in the duration of the activity; * represents statistical significance, *p* < 0.05.

### 3.5 CRF changes after isochronous substitution of MVPA

After adjusting for age, gender, place of residence, BMI, and SES, the study extended the substitution increment to 60 min, using 5-min intervals, to investigate the dose–response relationship between different substitution durations and CRF for each 24-h activity behavior that showed significant substitution effects (MVPA, SLP, SB, and LPA). The results indicated the following:

- As the time of MVPA replacing SB, SLP, and LPA increased, the predicted CRF value exhibited a continuous upward trend, with the increase being most pronounced for MVPA/SB, followed by MVPA/LPA, and then MVPA/SLP (Figure 2).
- The dose-effect relationship between the mutual substitution of MVPA and other behaviors and CRF was asymmetric. Specifically, as the time of MVPA replacing other behaviors increased, CRF would slowly increase, whereas the reverse substitution would cause CRF to decrease rapidly. For instance, when 30 min of MVPA replaced SB, CRF increased by 0.52 units, but when SB replaced MVPA for the same duration, CRF decreased by 0.84 units.
- When MVPA replaced LPA, SB, and SLP for 10 min, CRF increased by 0.13, 0.19, and 0.07 units, respectively. However, over the next 15–60 min, the rate of CRF increase gradually slowed, with increases ranging from 0.19 to 0.7, 0.22 to 0.90, and 0.13 to 0.69 units, respectively. Conversely, when LPA, SB, and SLP replaced MVPA for 10 min, CRF decreased by 0.17, 0.19, and 0.14 units, respectively. Over the next 15–60 min, the rate of CRF decrease accelerated, with decreases ranging from 0.26 to 5.47, 0.32 to 6.11, and 0.19 to 5.21 units, respectively. This result indicates that 10 min is the turning point in the health benefits of MVPA and other behaviors.

## 4 Discussion

Recently, Liang et al. (27) conducted a compositional analysis to examine the impact of reallocating different 24-h activity behaviors on physical health indicators in older adults, focusing primarily on blood pressure as a surrogate measure of cardiopulmonary function. Building on their findings, we leveraged VO2max, the gold-standard indicator of CRF, to provide a more comprehensive assessment of how the isocaloric substitution of various 24-h activity behaviors influences CRF and overall cardiopulmonary endurance in this population. This approach enables a more nuanced understanding of activity-related health outcomes in older adults. By analyzing the variation matrix, we identified the potential for mutual conversion between different activity behaviors. Specifically, MVPA time in the older adult appears relatively stable, showing the lowest likelihood of conversion to other behaviors. Conversely, the transition between SB and SLP is most frequent, likely reflecting the older adult’s adjustment of rest and activity times in response to their physical condition and daily needs. Notably, the transition between SB and MVPA is minimal, indicating that once personal lifestyle habits are established, they become resistant to change in old age. Furthermore, LPA time remains relatively stable, exhibiting limited conversion to other behaviors.

Analyzing 24-h activity behavior as an integrated whole revealed significant correlations between these activities and CRF. In contrast to patterns observed in children and adolescents, the relationships between moderate-to-vigorous physical activity MVPA, LPA, SB, SLP, and CRF in the older adult were distinct. Specifically, the proportion of time spent on MVPA showed a significant positive correlation with CRF, whereas SB was significantly negatively correlated with CRF. No significant correlations were observed between LPA, SLP, and CRF. To date, empirical studies utilizing component analysis to examine CRF are sparse. In China, only one study has applied this method to explore the relationship between 24-h activity behaviors and CRF in college students (31), with findings consistent with those of our study.

From a physiological standpoint, exercise training is a highly effective intervention for enhancing CRF. Our study confirmed that increasing MVPA time has a positive impact on CRF. MVPA enhances CRF by improving cardiac hemodynamics, optimizing energy metabolism pathways, and promoting endothelial function and angiogenesis. Specifically, MVPA improves myocardial contractility and ventricular loading capacity (32), increases stroke volume, thereby enhancing cardiac output and the body’s oxygen delivery capacity. Furthermore, MVPA activates the AMPK and SIRT1 pathways, upregulates PGC-1α expression, promotes fatty acid oxidation, and glucose uptake and utilization, thereby improving energy production efficiency (33). MVPA also increases shear stress, stimulates nitric oxide synthase (NOS) activity, elevates nitric oxide (NO) production, enhances vasodilation, reduces vascular resistance, increases capillary density, and ultimately improves tissue oxygenation and nutrient supply (34).

Conversely, sedentary behavior detrimentally affects CRF by promoting vascular dysfunction, diminishing muscle metabolic activity, and heightening the risk of metabolic syndrome (35). Prolonged sitting reduces shear stress, decreases nitric oxide production, impairs endothelial function, and elevates the risk of arteriosclerosis (35). Additionally, sedentary behavior disrupts muscle metabolic pathways, leading to reduced glucose sensitivity, induced insulin resistance, diminished mitochondrial function, decreased fatty acid *β*-oxidation, and lower energy metabolism efficiency (36). Sedentary behavior is also positively associated with components of metabolic syndrome (37), collectively contributing to a decline in cardiopulmonary function (38, 39).

While the direct impact of LPA on CRF may not be as pronounced as that of MVPA, previous studies have demonstrated that LPA contributes to the maintenance and slight enhancement of CRF levels by improving microvascular health, increasing muscle activity, and promoting blood flow redistribution. LPA enhances muscle activity, improves endothelial function through shear stress, and helps maintain vascular health. Furthermore, it redistributes blood flow from inactive tissues to metabolically active ones, thereby improving metabolic rate and energy efficiency, playing a supportive role in maintaining baseline cardiopulmonary function.

In summary, this study elucidates the mechanisms by which MVPA, SB, and LPA impact CRF in the older adult. It underscores the importance of health interventions that comprehensively address the appropriate allocation and substitution of different activities to optimize cardiopulmonary health, providing a scientific basis for designing effective health promotion strategies.

By employing the component isochronous substitution model, we predicted changes in CRF following the reallocation of 15 min among different elements of 24-h activity behavior. The results revealed that substituting MVPA for other activities, particularly for SB, significantly enhances CRF. Conversely, reducing SB time to increase MVPA or LPA also positively impacts the cardiopulmonary health of the older adult. These findings highlight the importance of increasing moderate or higher intensity physical activity and reducing sedentary time to maintain and improve cardiopulmonary endurance, consistent with previous research across different populations (38, 40).

In China, older adult individuals often experience increased leisure time post-retirement, leading to excessive sitting and a marked deficiency in basic physical activity and exercise. This sedentary lifestyle contributes to weakened bodily functions and a decline in cardiopulmonary fitness (12). The significant energy expenditure difference between MVPA and SB further accentuates the importance of substitution; when SB replaces other activities, it negatively impacts CRF, reinforcing the conclusion that reducing SB time in favor of increasing LPA or MVPA benefits health.

From the perspective of the interconnection between 24-h activity behaviors, MVPA remains relatively stable, with conversions primarily occurring between MVPA and LPA. Directly converting SB into MVPA is challenging; thus, mitigating SB’s adverse effects should focus on converting SB into LPA as much as possible. Additionally, the study observed significant changes in CRF when SLP replaced SB or LPA, and when LPA replaced SB. This result is not entirely consistent with existing studies, possibly due to varying cutoff values for physical activity intensity. This study utilized the cutoff points established by Evenson et al. (41), whereas other scholars have employed different SB/LPA intensity cutoffs based on accelerometer data, leading to potential discrepancies. A lower SB/LPA intensity cutoff may obscure the distinction between SB and LPA, complicating the assessment of their health benefits.

This study also conducted a dose-effect analysis on the impact of 24-h activity behavior elements with significant substitution effects (e.g., mutual substitution of MVPA and other behaviors) on CRF. The results indicated an asymmetry in CRF changes: when MVPA was replaced by other activities, CRF increased slowly; conversely, when other activities replaced MVPA, CRF decreased rapidly. This phenomenon may be attributed to two factors. First, the substantial time base difference between activities—wherein the average daily sitting time for this group was about 10 h, while total MVPA time was less than 1 h—means that reallocating 15 min from MVPA to other behaviors represents a significant substitution effect, whereas reallocating 15 min from SB has a negligible impact.

Second, the health benefits of physical activity (PA) accumulate over time. Continuous PA engagement leads to sustained health benefits, but reducing or discontinuing PA results in a rapid decline in these benefits. Building on Liang et al. (27) analysis of 24-h activity reallocations and physical fitness in older adults, our study specifically examines VO2max to highlight the effects of isocaloric substitutions between activity types. We identify a 10-min threshold as an optimal point for substituting MVPA, offering insights into the dose-dependent impacts of activity modifications on cardiorespiratory health in this population. According to the dose-effect curve, after MVPA replaced LPA, SB, and SLP for 10 min, the growth rate of CRF gradually slowed, suggesting that on the current MVPA activity baseline (49.91 min/day), adding 10 min of MVPA per day—totaling approximately 60 min/day—yields the most substantial improvement in CRF. This finding aligns with the recommendation of 60 min of physical activity per day for the older adult in China. Therefore, it is advisable to transform SB into MVPA as much as possible during 10-min breaks to promote the enhancement of cardiopulmonary health in the older adult. This study has several limitations. First, its cross-sectional design restricts the ability to infer causality between 24-h activity behaviors and CRF. Second, the sample was drawn solely from older adults in Tianjin, China, potentially limiting the generalizability of the findings to other populations. Additionally, although a 3D accelerometer was used to objectively measure physical activity, it could not capture non-wear time and distinguish some low-intensity behaviors, possibly underestimating certain activities. One major limitation is that the researchers, particularly ask the participants worn the accelerometer on the right hip, being a better protocol to address these limitations wrist-worn protocols (42). Moreover, while compositional data analysis effectively addresses collinearity, it may not fully capture the complex interactions between behaviors. Future longitudinal studies with broader samples and more refined measurement tools are needed to validate these findings.

## 5 Conclusion

This study, which examined the relationship between 24-h activity behavior and CRF in 540 older adult individuals in Tianjin, confirmed the hypothesis that MVPA is significantly positively correlated with CRF, while SB is significantly negatively correlated with CRF. These findings provide new evidence for the management of cardiopulmonary health in the older adult. The study highlights the importance of adjusting daily activity patterns—specifically by increasing MVPA and reducing SB time—as crucial strategies for improving CRF. The application of the component isochronous substitution model further elucidates the quantitative impact of substitutions between different activity behaviors on CRF, offering a concrete basis for health guidance in the older adult population.

These results have practical implications for designing effective health promotion programs, suggesting that simple behavioral adjustments in daily life can significantly benefit the cardiopulmonary health of older adults. Future research should consider the influence of individual differences, such as gender and baseline health conditions, on the relationship between activity behavior and CRF to develop more tailored health interventions. Furthermore, with ongoing advancements in technology, the use of sophisticated monitoring tools to conduct detailed analyses of older adult activity behavior will deepen our understanding of the relationship between activity behavior, CRF, and other health indicators. This will, in turn, provide a more comprehensive scientific foundation for promoting the overall health of the older adult.

## Data Availability

The raw data supporting the conclusions of this article will be made available by the authors, without undue reservation.
